# Novel Compound Heterozygous Variants in the *LHCGR* Gene in a Genetically Male Patient with Female External Genitalia

**DOI:** 10.4274/jcrpe.galenos.2018.2018.0197

**Published:** 2019-05-28

**Authors:** Mei Yan, Julaiti Dilihuma, Yanfei Luo, Baoerhan Reyilanmu, Yiping Shen, Maimaiti Mireguli

**Affiliations:** 1 First Affiliated Hospital of Xinjiang Medical University, Department of Pediatrics, Xinjiang Uygur Autonomous Region, China; 2 Boston Children’s Hospital Harvard Medical School, Department of Genetics and Genomics, Massachusetts, United States

**Keywords:** Disorder of sexual differentiation, Leydig cell hypoplasia, LHCGR gene, novel variants

## Abstract

The *LHCGR* gene encodes a G-protein coupled receptor that plays a pivotal role in sexual differentiation in males, ovarian development in females and in fertility via its interaction with luteinizing hormone and chorionic gonadotropin. Inactive variants of the *LHCGR* gene cause Leydig cell hypoplasia (LCH), which is a rare disease and one of the causes of disorder of sexual differentiation (DSD) in males. The aim of this work was to clarify the clinical and molecular characteristics of a 2.75 year old patient with type 1 LCH. Whole exome sequencing was performed for the patient family and variants in the *LHCGR* gene were validated by Sanger sequencing. Pathogenicity of the missense variant was evaluated by multiple *in silico* tools. Our Chinese patient, who exhibited DSD, had female external genitalia (normal labia majora and minora, external opening of urethra under the clitoris and blind-ended vagina) and bilateral testis tissues in the inguinal region. Genetic sequencing revealed compound heterozygous variants in the *LHCGR* gene in the patient, including a novel missense variant in exon 4 (c.349G>A, p.Gly117Arg) and a novel nonsense variant in exon 10 (c.878C>A, p.Ser293*). The missense variant is in the first leucine-rich repeat domain of the *LHCGR* protein, which is predicted to affect ligand recognition and binding affinity and thus protein function. The patient is molecularly and clinically diagnosed with type 1 LCH, which is caused by novel, compound heterozygous variants of the *LHCGR* gene. We believe this report will serve to expand the genotypic spectrum of *LHCGR* variants.

**What is already known on this topic?**

Both loss and gain of function mutations of the *LHCGR* gene can cause human diseases. Inactive *LHCGR* variant causes type 1 Leydig cell hypoplasia, which is characterized by the complete absence of male differentiation. To date, 77 variants have been reported, including 49 missense, 11 nonsense, five gross deletions, four small insertions, four small deletions, three splicing variants and one gross insertion.

**What this study adds?**

In this study, we identified two novel heterozygous variants in the *LHCGR* gene (c.349G>A, p.Gly117Arg and c.878C>A, p.Ser293*) causing type 1 Leydig cell hypoplasia in a 2.75 year old patient presenting with female external genitalia and bilateral testis tissue in the inguinal region.

## Introduction

The human luteinizing hormone (LH)/chorionic gonadotropin (CG) receptor (*LHCGR*; OMIM #52790) gene belongs to the G-protein coupled receptor 1 family. The *LHCGR* gene encodes a shared receptor for both LH and CG and the receptor plays a critical role in male sexual differentiation, female ovarian development and fertility (1). *LHCGR* is located on chromosome 2p21 and contains 12 exons. The *LHCGR* gene encodes a 699 amino acid protein that consists of an N-terminal cysteine-rich region, a tandem leucine-rich repeats (LRRs) region and a C-terminal cysteine-rich region (2,3). In males, the N-terminal region and the LRR1-LRR7 repeats are essential for the high affinity binding of human CG (hCG), which stimulates the production of testosterone and maturation of fetal Leydig cells during early embryogenesis. In addition, the interaction between LH and *LHCGR* maintains a postnatal testosterone level that is required for male secondary sex characteristics and spermatogenesis during puberty (4,5).

Both loss and gain of function mutations of the *LHCGR* gene can cause human diseases. In males, germline activation of *LHCGR* is associated with inherited, autosomal dominant precocious puberty (OMIM#152790). Biallelic inactivation of the *LHCGR* causes Leydig cell hypoplasia (LCH, OMIM#238320) that leads to male disorders of sexual differentiation (DSD). Constitutively inactive *LHCGR* variant causes type 1 LCH, which is characterized by the complete absence of male differentiation. Partially inactive *LHCGR* variants result in type 2 LCH that features hypogonadal phenotypes with variable severity (6,7). In females, inactivated *LHCGR* gene has no effect on the primary and secondary sex characteristics, but it causes amenorrhoea and infertility due to aberrant follicular maturation and ovulation (8).

In this study, we report a rare pediatric patient of type 1 LCH due to novel, compound heterozygous mutations in the *LHCGR* gene. Our findings expanded the spectrum of genotype-phenotype correlation in the *LHCGR* variants.

## Case Report

The proband was a 2.75 year old child whose social gender was female. The child was taken to our hospital due to absence of vagina. The patient was born full term by spontaneous delivery, and she is the second child of healthy parents of non-consanguineous marriage. Her birth weight was 3,900 g. Her weight at presentation was 17 kg (96.8^th^ percentile) and her height was 97 cm (73.5^th^ percentile). Physical examination showed that the patient exhibited predominantly female external genitalia, with normal bilateral labia majora, bilateral labia minora and external opening of urethra under the clitoris. However, she had a blind-ended vagina without external opening. The patient showed absence of scrotum and penis. Abdominopelvic ultrasound examination detected bilateral testis tissues in the inguinal region (left 2.0 cm×0.7 cm×0.9 cm; right 1.7 cm×0.7 cm×0.9 cm). Uterus or other Mullerian structures were not observed. Laboratory results showed that the patient had extremely low serum testosterone and dihydrotestosterone levels (0.01 nmol/L), which could not be stimulated by hCG. Serum levels of LH and follicle stimulating hormone were within the normal ranges (3.84 IU/L and 9.09 IU/L, respectively) and both of were hyper-responsive (24.48 IU/L and 22.33 IU/L, respectively) to stimulation with 2.5 µg/kg of LH releasing hormone. Thyroid hormones, estradiol, prolactin, blood chemistry and complete blood count were all normal. Primary genetic analysis revealed that the patient’s karyotype was 46, XY and no pathogenic variant was identified in the *SRY* gene. The patient was primarily diagnosed as a case of male pseudohermaphroditism.

All procedures followed were in accordance with the ethical standards of the responsible institutional committee on human experimentation and with the Helsinki Declaration of 1975, as revised in 2000, and the protocol was approved by the Ethics Committee of the First Affiliated Hospital of Xinjiang Medical University (approval no: XJMU-FAHIRB-2017005). Informed consent was obtained from the patient’s family.

### Genetic Sequencing

To obtain a rapid and accurate clinical genetic diagnosis, trio-whole exome sequencing (WES) was used to screen for causal variants. Briefly, a total of 3 µg of genomic DNA was sheared to obtain DNA fragments with sizes between 150 bp and 200 bp. The capture library was prepared using SureSelect Human All Exon V6 kit (Agilent Technologies Inc., Santa Clara, CA, US) following the manufacturer’s protocol. Next, clusters were generated by isothermal bridge amplification with an Illumina cBot station and sequencing was performed by an Illumina X10 System (Illumina, CA, USA). Alignment of sequence reads to the reference human genome (Human 37.3, SNP135) was performed using the NextGENe® software (SoftGenetics, PA, USA). All single nucleotide variants (SNVs) and indels were saved in a VCF format file, which was then uploaded to Ingenuity^®^ Variant Analysis™ (Ingenuity Systems, CA, USA) for biological analysis and interpretation. The variants were validated by Sanger sequencing using the ABI3730XL sequencer (Applied Biosystems, Thermo Fisher Scientific, Inc., Waltham, MA, USA) with the forward and reverse primers. The potential pathogenicity of the missense variant was analyzed by using MultAlin (http://multalin.toulouse.inra.fr/multalin/), PolyPhen-2 (http://genetics.bwh.harvard.edu/pph2/), Combined Annotation Dependent Depletion (CADD) (http://cadd.gs.washington.edu/), and MutationTaster (http://www.mutationtaster.org/).

### Identification of the Causal Variants

**Figure 1:**
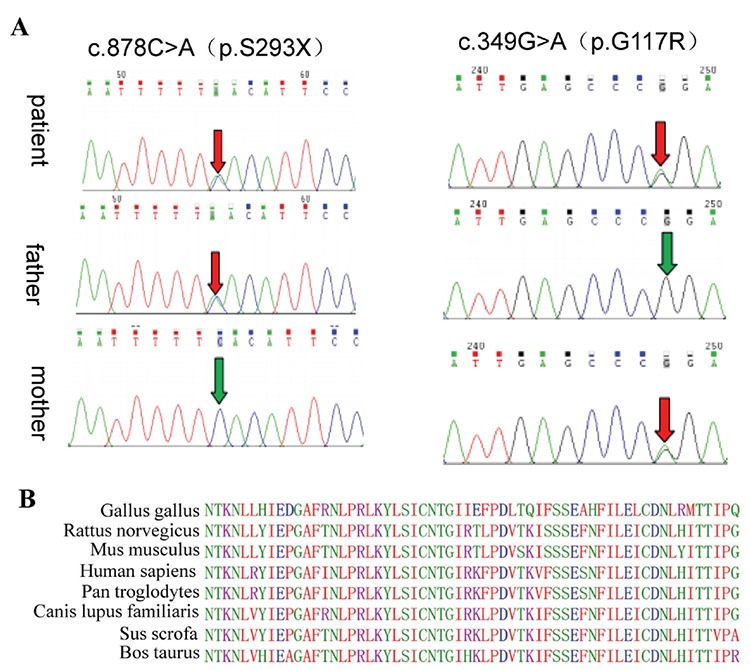
Genetic sequencing of the *LHCGR* gene. (A) Sanger sequencing confirmed a novel heterozygous missense variant in exon 4 (c.349G>A, p.Gly117Arg) and a novel heterozygous nonsense variant in exon 10 (c.878C>A, p.Ser293*) in the patient, which were inherited from the parents. (B) The referred amino acid of codon 117 (Gly) is highly evolutionarily conserved across species

For the patient, WES yielded a total of 103,509,228 reads, and the mean target coverage was 133 reads with 95.52% having 20× coverage and 99.83% having 1× coverage. The candidate variants were first filtered by the following parameters: (1) minor allele frequency (MAF) under 1% in genome Aggregation Database (gnomAD, http://gnomad.broadinstitute.org/); (2) the benign variants, including synonymous and harmless missenses predicted by Ingenuity and those predicted to have no impact on splicing by MaxEntScan. Subsequently, clinical symptoms of male pseudohermaphroditism were used as filtering indexes to analyze the candidate variants. As a result, we identified a compound alteration with two heterozygous variants within the *LHCGR* gene, which we believe to have contributed to the patient’s condition. Of the two variants, one is a novel missense variant in exon 4 (c.349G>A, p.Gly117Arg), and the other was a novel nonsense variant in exon 10 (c.878C>A, p.Ser293*). We have further confirmed the compound heterozygous variants by Sanger sequencing. The patient’s father was heterozygous for the nonsense variant and the patient’s mother was heterozygous for the missense variant (Figure 1A).

### Pathogenicity Predictions for c.349G>A (p.Gly117Arg)

**Figure 2:**
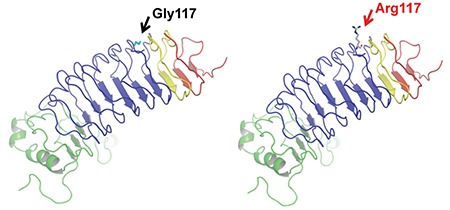
Three-dimensional structure model of the *LHCGR* protein. The indicated amino acid (p.117, colored arrow: black, wild-type; red, variant) is located in the first leucine-rich repeat domain of the *LHCGR* protein

To evaluate the pathogenicity of the novel variant c.349G>A, we first analyzed the conservation of Gly117 using MultAlin software. As shown in Figure 1B, results from MultAlin show that the amino acid glycine at codon 117 is highly evolutionarily conserved. Next, we used three *in silico* prediction software analyses to evaluate the impact of the variant on protein function. The PolyPhen-2 score of the variant is 0.96, indicating that the variant is probably damaging. The MutationTaster score is 1, which implies that the variant is likely disease causing. The CADD score is 25.4, which suggests that the variant can be damaging. To better understand the missense variant, the WT and variant amino acid at codon 117 were modeled into the three-dimensional structure of the *LHCGR* protein (9) (Figure 2). Based on the structure (9) and domain information of the *LHCGR* wild-type protein obtained from Uniprot (http://www.uniprot.org/), the amino acid substitution at the 117th position *(p.Gly117Arg)* was predicted to disrupt the first LRR domain, which may affect recognition and binding affinity of *LHCGR* to hCG and/or other ligands. Taken together, our analysis results indicate that the *c.349G>A (p.Gly117Arg)* variant is likely harmful to the protein function.

## Discussion

In the current study, we report a socially defined female, Chinese patient presenting with a DSD. The patient had normal labia majora and minora, external opening of urethra under the clitoris, but a blind-ended vagina, a karyotype of 46, XY and bilateral testis tissues in the inguinal region. By performing WES, we identified a compound heterozygous variant in the patient, with a novel missense variant (c.349G>A, p.Gly117Arg) and a novel nonsense variant (c.878C>A, p.Ser293*) in her *LHCGR* gene that contributed to the patient’s condition. The missense and nonsense variants were inherited from the unaffected heterozygous father and mother, respectively. According to the variant interpretation guidelines from the American College of Medical Genetics and Genomics/the Association for Molecular Pathology (10), the nonsense variant is classified to be pathogenic (PVS1 + PM2 + PP4), and the missense variant is also likely to be pathogenic (PM2 + PM3 + PP3 + PP4). Therefore, the patient was molecularly and clinically diagnosed with type 1 LCH.

**Figure 3:**
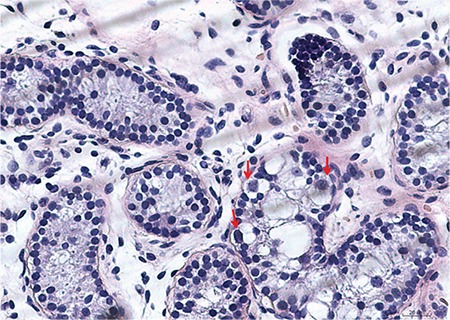
Histologic analysis of the surgical testicular tissue samples (400x). Hematoxylin and eosin staining revealed that the seminiferous tubules were lined only by a few Sertoli cells, and the interstitial tissue appeared to have only a few fusiform cells that might be immature Leydig cells

Gender assignment for LCH patients is influenced by genital appearance, surgical options, fertility potential and the views of the family, and can be difficult (11). Timing of gender assignment can also be controversial, especially when the psychological age is taken into consideration. The social gender of our patient was female. We assessed that the patient’s psychological gender was also female. Physically, the patient’s abnormal testis tissues showed no function in the provocation test using hCG. Based on medical advice from experts and discussions with the parents, the patient underwent bilateral orchidectomy. Testicular histology revealed that the seminiferous tubules were lined only by a few Sertoli cells. The interstitial region appeared to have only a few fusiform cells that appeared to be immature Leydig cells (Figure 3), a finding which is consistent with LCH phenotypes and confirmed the diagnosis of LCH. Interestingly, several previously reported cases showed that delayed orchidectomy after adolescence might result in primary amenorrhea and breast underdevelopment (12,13). For these reasons, orchidectomy was performed in our patient right after the gender assignment, and normal development of secondary female characteristic is expected in the future.

To date, a total of 77 variants have been identified in the *LHCGR* gene (Human Gene Mutation Database: http://www.hgmd.cf.ac.uk/), including 49 missenses, 11 nonsenses, five gross deletions, four small insertions, four small deletions, three splicing variants and one gross insertion. In contrast to the infrequency of activating variants, inactivating homozygous and compound heterozygous variants that alter structure of the *LHCGR* protein and subsequently its function is more common. As shown in Table 1, we summarized the inactivating variants from the reports in the literature (14,15,16,17,18,19,20,21,22,23,24,25). Interestingly, although the frequency of these variants is extremely low in gnomAD database (most of them are 0), homozygous variants account for most gene variations in the patients. Variants occurring more frequently in exon 11 may be simply explained by the fact that it is the largest exon of the *LHCGR* gene.

In our case, the nonsense variant (p.Ser293*) is a loss of function mutation, and our analysis shows that the missense variant (p.Gly117Arg) is mostly likely also a loss of function mutation, which lead to the inactivation of *LHCGR*. However, the functional analysis is lacking and this should be performed in a future study.

We report a 46, XY, DSD Chinese Uyghur patient with type 1 LCH with novel heterozygous compound variants in the *LHCGR* gene. Her clinical features correlated with the molecular diagnosis. She was treated after choosing her social gender to be female. This is one of only a few LCH cases that underwent gender assignment and treatment following molecular confirmation of clinical diagnosis.

## Figures and Tables

**Table 1:**
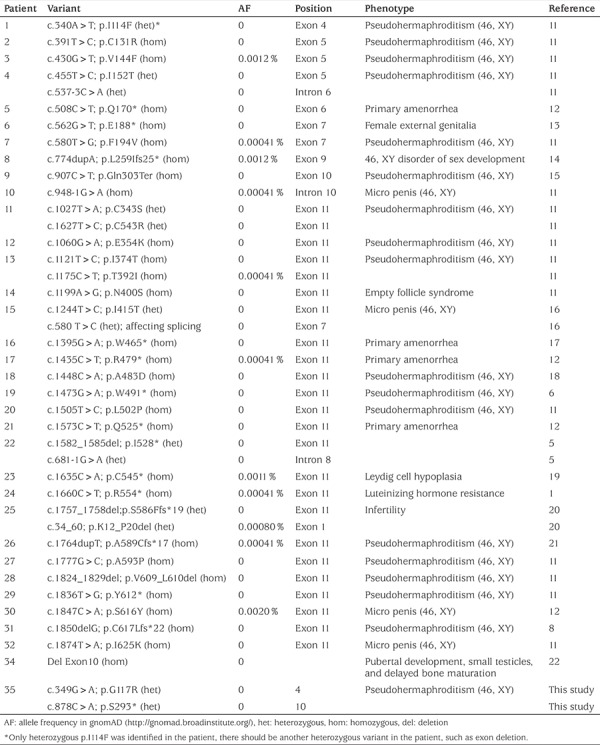
A summary of the inactivating variants of *LHCGR* gene
